# Beyond Organisational Borders: The Soft Power of Innovation in the Health Sector: Comment on "What Managers Find Important for Implementation of Innovations in the Healthcare Sector – Practice Through Six Management Perspectives"

**DOI:** 10.34172/ijhpm.2022.7270

**Published:** 2022-07-06

**Authors:** Cátia Miriam Costa

**Affiliations:** Instituto Universitário de Lisboa (ISCTE-IUL), Centro de Estudos Internacionais, Lisbon, Portugal.

**Keywords:** Health, Innovation, Internationalisation, Diplomacy, Knowledge Transfer, Science and Technology

## Abstract

Health is not just a physiological state, it is also a relational phenomenon. This means health is a collective challenge, often a cross-border one. Diplomacy in the health sector has progressively received more attention from formal actors (national states, international organisations, etc) but after the coronavirus disease 2019 (COVID-19) challenge, this attention became a global emergency mobilising an expansive set of knowledge-seeking players (industry, research networks, civil society, etc). This paper comments on and leverages the contribution by Palm and Feschier on innovation management at the organisational level to address a complementary dimension: the internationalization process, and the need for a particular set of skills and routines to make innovations travel through different markets and regulatory contexts. Our argument is that marketing (knowing about customers) and diplomacy (understanding framing institutions) constitute a set of dynamic capabilities (soft power) that are critical for the effective internationalization of innovation.

## Introduction

 The health sector is an expansive realm. As a service sector, it is a heavy user of science and technology, it integrates a high number of inputs from a variety of knowledge specialisms as treatments and solutions become more complex, and it typically grows at a rhythm that is higher than gross domestic product. Moreover, with the pandemic that exploded in early 2020 as a global emergency, health challenges became a synchronous international puzzle for public actors (states and international organisations) and private players (companies, universities, non-governmental organisations, etc). This international dimension, with the need for cross-cultural understanding and mutual adjustment (between different health system traditions, specific regulatory cultures, etc), constitutes the focus of this piece.

 For decades, health diplomacy was centred on state cooperation under the World Health Organization (WHO) umbrella and was predicated on the universalisation of access to health. Knowledge transfer (that is, the out-building of the capabilities underpinning new products and processes) was not the central problem due to the difficulties faced by low-income countries and the fact was that most of the know-how was concentrated in the developed world. Only after some developing countries started to have the ability to develop an autonomous health sector (pharmaceutics, healthcare, etc) did the debate start to encompass genuine multilateral cooperation for innovation. In turn, the affirmation of new geographies resulted in the recognition that specific innovations could be helpful, of interest and mainly applicable to countries of the Global South.

 Since some time ago, companies have pushed along internationalisation pathways in the health sector. However, healthcare internationalisation is more challenging, as the rules are precisely cut to the home base and obey national institutions’ determinations (eg, rules, certification, etc). Moreover, internationalising innovation in the health sector implies the introduction of solutions and protocols which undergo thorough scrutiny in each domestic market. Internationalisation is thus a slow, costly, and uncertain process. Therefore, the issue is making internationalising healthcare innovation more manageable.

 In their recent article, Palm and Fischier^1^ note a growing expectation that many health organisations will innovate. However, according to these authors, the implementation phase is where there are more difficulties in practice. They offer a review of a set of enabling factors that can facilitate the movement from idea generation to implementation in the healthcare context. We problematise the (international dimension of the) healthcare context and extend their view. Our research question: what can a relational component add effective cross-border/cross-organisational innovation strategies? We argue that marketing (knowing about customers) and diplomacy (understanding framing institutions) constitute a set of dynamic capabilities (soft power) that are critical for the effective internationalisation of innovation. Building expertise at the intersection of industry and diplomacy is crucial for a more robust and resilient health ecosystem, which is in itself a societal leverage for coping with a more uncertain and fragmenting world.

## Implementing Innovation in the Healthcare Sector

 Palm and Fischier^1^ have carried out a study in which they point out that health sector managers should try to enhance their attitude towards innovations and create leeway for realist implementation. They inquire about what can facilitate moving from idea generation to implementation in a healthcare context. For this, they advocate a holistic approach comprised of six factors, namely (*i*) active collaboration with the beneficiaries of healthcare (ie, patients as innovation users), (*ii*) cooperation with relevant stakeholders (eg, universities), (*iii*) organisational culture (ie, shared vision and social norms at the company level), (*iv*) human resource management (eg, through continuous collective learning), (*v*) organisational structure (ie, allowing for small teams and fast sprints), and (*vi*) resource availability (adaptive cost control).

 Through realistic and reflexive “concretisation,” we are told, managers can realize a more “practically oriented” approach and achieve the sustained delivery of healthcare outcomes. Rather than stating a one best way configuration to any given health subsector, Palm and Fischier are cautious and flexible enough and suggest the importance of tailoring innovation strategies based on “each organization’s unique contextual conditions.” That is, to say, effective implementation of innovation needs proper handling, adaptation to each unique context, and a sense for the good conditions that align to facilitate industrial and commercial progress in the field of health.

 Palm and Fischier caution against mere attitudinal dispositions toward innovation (managerial “lip service”) and instead invite the actual effort to set up the conditions for innovation to roll out (“creating structural space”). They end their contribution by calling for the “continued research should be conducted on how implementation theories can be concretised in different healthcare contexts.” In our take, we welcome their realist and reflexive plea and seek to extend their work by focusing on the international implementation of innovation. Indeed, going beyond the domestic setting implies another layer of complexity and situational effectiveness for innovation strategy.^2,3^

 At the intersection of internationalisation and innovation, there is the need to “create structural space” for manoeuvring and leveraging knowledge for the sake of implementing new value propositions in different healthcare contexts. We argue that cross-national healthcare operations are contingent upon marketing and diplomatic capabilities. This set of “soft power” assets and activities allow for the opening up and sustainability of innovation-intensive international business in what is a highly-regulated and idiosyncratic healthcare ecosystem.^4^

 Carving structural space for healthcare innovations while performing international movement goes beyond technical knowledge regarding health products and processes. The cross-border rollout of healthcare innovation implies a capacity to deal with different clinical and regulatory cultures, certification traditions, solution-absorption templates. The key for unlocking the potential of international innovation is, we argue, the projection of “soft power.”

## Developing and Deploying “Soft Power” in the Innovative Internationalised Health Sector

 By “soft power,” we refer to the compact of relational skills and repertoires that can be deployed at the micro-level (ie, commercial negotiations and contracting) and the macro-level (ie, institutional coordination and bureaucratic validation). However, theoretically applied at a macro-level, soft power is usually defined as the capacity to directly influence others in the direction one wants, building attraction to the orientations given by someone.^5^

 Although conceptualised for scenarios of international politics, soft power is also applicable at the micro-level, including to companies and small and medium entreprises, business communities and regional hubs.^6^ Soft power can contribute to deepercooperative relationships providing conditions for international expansion or attracting local partners to its business.^7^ When this cooperation or partnership occurs in markets with different levels of development, this can help a firm’s internationalisation and provide the target-market advanced technologies and the upskilling of human resources, benefiting the human capital and the creation of new networks.

 In the health sector, the deployment of soft power went through a dynamic and evolutive process that began with the product transference through marketing and skilling of human resources to use these products. In general, health diplomacy much contributed to the transfer of the products for the global south (a prime example of which are WHO working groups, in these fora academia, industry and non-governmental organisations have a key role in providing evidence for international resolutions on specific matters). However, bringing processes into this “transfer” rationale happened later and was often based on private or public companies, hospitals and laboratories enhancing the soft internationalisation of the health sector by attracting local partners to these new techniques and skills.

 Although facing the challenge of solid regulation parameters and certification procedures, the health sector firms managed to internationalise through innovation and adaptation to different markets and other needs. The shaping of structural spaces implied interaction with companies and authorities, which reflected the merging of diverse diplomacies and the convergence of different agendas. Combining health diplomacy with scientific, technological, and innovation diplomacy (the latter more adapted to commercial objectives and gathering public and private stakeholders) enhanced this new trend in the international health market.^8^

## Shaping the Structural Space: Companies

 Innovative firms tend to be more successful in international business, and outwardness puts them in contact with alternative cultures and selective environments, thus adding to their overall knowledge base.^9^ In the global marketspace, things happen regionally and sectorally. Firms are thick organisations with specific transmission mechanisms that operate via internal networks. Nevertheless, they are also place-based communities, and knowledge flows through the contacts in the local environments in which the units operate.

 Health science has become an ever globally distributed endeavour, so has innovation. Not only has the relocation of multinational corporation research and development reshuffled the international distribution of intellectual labour, but complexity has risen too: value chains have become longer while the ability to commercially exploit discoveries have become more dependent on local expertise.

 Systemic innovation is crucial in the health sector. Therefore, encouraging and enabling interactive learning among public, private, and non-governmental players is a crucial pathway for innovation.^10^ The private actors include companies, both large (system builders) and small (specialised suppliers), with wide-ranging contribution levels to the knowledge ecosystem. These actors develop, co-develop or identify external resources to help secure and scale promising opportunities while finding ways to help accelerate the commercialisation of innovation through a close negotiation with national regulations, which are themselves correlated with international standards and conventions.^11^

 In the light of the many facets of innovation, the ability to enter and navigate local/national (but internationalised) sectoral systems is therefore of the essence. In the sectoral health system, marketing and commercial capabilities that allow for the understanding and persuasion of the “other” is called for. They matter whether the international presence is simpler, such as through exports, riskier, such as the establishment of production subsidiaries and the development of cooperation with research-based partners.^12^

## Shaping the Structural Space: Authorities

 Science and technology diplomacy is not equally nurtured everywhere.^13^ With the idea that innovation has become commonplace in public policy, it would seem that innovation communication would be perceived as equally important.^14,15^ But, no. Soft power, which relies on the positive public image carried by knowledge prowess on the world stage, is a scarce asset.

 Not all countries invest a lot in international relations, and not all invest enough in research and development. As an overlapping category, healthcare innovation diplomacy is built at the level of discourse and the level of action.^16^ It is a hybrid category since it refers to a diversity of actors and partners: research and regulatory institutions (that is, knowledge and normative authorities) and a range of other actors, from professional associations to industrial associations.

 Health innovation diplomacy should be a new combination: of the fields of international relations (with its orientation on power) and health policy (with its orientation on health opportunities and science).^11^ Moreover, in the discourse-base era, in which reputation and branding are critically complementary assets to technical knowledge, attracting the attention and good-will of partners and framing institutions is of the essence as means to advance effective innovation strategies.^17,18^

## Conclusion

 In a world with new polarities and in which health system stakeholders are increasingly knowledgeable (including patients), an understanding of relational dynamics matters increasingly. The importance of the soft side of the innovation powers need to deal with societal challenges and industrial competitiveness should not be underestimated. In this paper, we argue for the need to foster marketing and communication capabilities (complementary leverages to those factors already highlighted by Palm and Feschier^1^) in the implementation process of innovation, especially across organisational boundaries and geographical borders.

## Ethical issues

 Not applicable.

## Competing interests

 Author declares that she has no competing interests.

## Author’s contribution

 CMC is the single author of the paper.

## Funding

 The Foundation for Science and Technology of the Portuguese Republic founded this research through the SFRH/BPD/113289/2015 project.

## References

[1] Palm K, Persson Fischier U (2022). What managers find important for implementation of innovations in the healthcare sector–practice through six management perspectives. Int J Health Policy Manag.

[2] Castellaci F, Grodal S, Mendonça S, Wibe M (2005). Advances and challenges in innovation studies. J Econ Issues.

[3] Caraça J, Lundvall BÅ, Mendonça S (2009). The changing role of science in the innovation process: from Queen to Cinderella?. Technol Forecast Soc Change.

[4] Costa CM, Mendonça S (2019). Knowledge-intensive consumer services Understanding KICS in the innovative global health-care sector. Res Policy.

[5] Wilson EJ (2008). Hard power, soft power, smart power. Ann Am Acad Pol Soc Sci.

[6] Ittelson P, Mauduit JC. Science & Diplomacy: How Countries Interact with the Boston Innovation Ecosystem. Washington, DC: DiploFoundation; 2019.

[7] Yu X, Si S (2012). Innovation, internationalization and entrepreneurship: a new venture research perspective. Innovation.

[8] Griset P (2020). Innovation diplomacy: a new concept for ancient practices?. Hague J Dipl.

[9] Filippetti A, Frenz M, Ietto-Gillies G (2011). Are innovation and internationalization related? An analysis of European countries. Ind Innov.

[10] Santos AB, Bogers ML, Norn MT, Mendonça S (2021). Public policy for open innovation: opening up to a new domain for research and practice. Technol Forecast Soc Change.

[11] Leijten J (2017). Exploring the future of innovation diplomacy. Eur J Futures Res.

[12] Freixanet J, Rialp A, Churakova I (2020). How do innovation, internationalization, and organizational learning interact and co-evolve in small firms? A complex systems approach. J Small Bus Manag.

[13] Varela C, Costa CM, Godinho MM. Diplomaciacientífica: do conhecimentoacadémicoao soft power político. Universidade Autónoma de Lisboa; 2017. p. 60-61.

[14] Costa CM (2015). Internacionalizaçãocomocontexto para novaspolíticas de ciência e tecnologia. ParceriasEstratégicas.

[15] Mendonça S. Diplomaciacientífica e tecnológica, comunicação de inovação e empreendedorismo. Universidade Autónoma de Lisboa; 2016. p. 46-47.

[16] Costa CM. The words of the belt and road initiative: a Chinese discourse for the world? In: Leandro FJ, Duarte PA, eds. The Belt and Road Initiative: An Old Archetype of a New Development Model. Singapore: Palgrave Macmillan; 2020. p. 23-44. 10.1007/978-981-15-2564-3_2.

[17] Castaldi C, Mendonça S (2022). Regions and trademarks: research opportunities and policy insights from leveraging trademarks in regional innovation studies. Reg Stud.

[18] Mendonça S, Damásio B, Charlita de Freitas L, Oliveira L, Cichy M, Nicita A (2022). The rise of 5G technologies and systems: a quantitative analysis of knowledge production. Telecomm Policy.

